# Transcriptomic signatures of peroxisome proliferator-activated receptor α (PPARα) in different mouse liver models identify novel aspects of its biology

**DOI:** 10.1186/1471-2164-15-1106

**Published:** 2014-12-15

**Authors:** Ewa Szalowska, Haftu A Tesfay, Sacha A F T van Hijum, Sander Kersten

**Affiliations:** RIKILT - Institute of Food Safety, Wageningen UR, P.O. Box 230, 6700 AE Wageningen, The Netherlands; Nutrition, Metabolism and Genomics Group, Wageningen University, Bomenweg 2, 6703 HD Wageningen, The Netherlands; CMBI Centre for Molecular and Biomolecular Informatics, Bacterial Genomics Group, Radboudumc, PO Box 9101, 6500 HB Nijmegen, The Netherlands

**Keywords:** Peroxisome proliferator-activated receptor alpha (PPARα), Fibrates, Lipid metabolism, Inflammation, Parenchymal and non-parenchymal liver cells, Mouse liver models, Transcriptomics

## Abstract

**Background:**

The peroxisome proliferator-activated receptor alpha (PPARα) is a ligand-activated transcription factor that regulates lipid catabolism and inflammation and is hepatocarcinogenic in rodents. It is presumed that the functions of PPARα in liver depend on cross-talk between parenchymal (hepatocytes) and non-parenchymal (Kupffer and endothelial cells) fractions as well as inter-organ interactions. In order to determine how cellular composition and inter-organ interactions influence gene expression upon pharmacological activation of PPARα, we performed a meta-analysis of transcriptomics data obtained from mouse hepatocytes (containing only the parenchymal fraction), mouse liver slices (containing both fractions), and mouse livers exposed to a PPARα agonist. The aim was to obtain a comprehensive view of common and model-specific PPARα-dependent genes and biological processes to understand the impact of cross-talk between parenchymal and non-parenchymal fractions as well as the effect of inter-organ interactions on the hepatic PPARα transcriptome.

To this end we analyzed microarray data of experiments performed in mouse primary hepatocytes treated with the PPARα agonist Wy14643 for 6 or 24 h (*in vitro*), mouse precision cut liver slices treated with Wy14643 for 24 h (*ex vivo*), and livers of wild type and Ppara knockout mice treated with Wy14643 for 6 h or 5 days *(in vivo)*.

**Results:**

In all models, activation of PPARα significantly altered processes related to various aspects of lipid metabolism. In *ex vivo* and *in vivo* models, PPARα activation significantly regulated processes involved in inflammation; these processes were unaffected in hepatocytes. Only *in vivo* models showed significant regulation of genes involved in coagulation, carcinogenesis, as well as vesicular trafficking and extracellular matrix.

**Conclusions:**

PPARα-dependent regulation of genes/processes involved in lipid metabolism is mostly independent of the presence of non-parenchymal cells or systemic factors, as it was observed in all liver models. PPARα-dependent regulation of inflammatory genes requires the presence of non-parenchymal cells, as it was observed only *ex vivo* and *in vivo*. However, the full spectrum of PPARα biology at the level of lipid metabolism, immunity, carcinogenesis, as well as novel aspects of PPARα signaling such as coagulation, vesicular trafficking and the extracellular matrix, seems to require systemic factors, as it was observed exclusively *in vivo*.

**Electronic supplementary material:**

The online version of this article (doi:10.1186/1471-2164-15-1106) contains supplementary material, which is available to authorized users.

## Background

Peroxisome proliferators, which include insecticides, herbicides, surfactants, phthalates, organic solvents, and hypolipidemic/anti-inflammatory fibrate drugs, were discovered as chemicals that upon administration to rodents increase the number of liver peroxisomes, stimulate fatty acid catabolism, and after chronic exposure cause hepatomegaly and hepatocarcinogenesis [1]. The effects of peroxisome proliferators are mediated by the peroxisome proliferator-activated receptor α (PPAR α) [2]. PPARα, together with PPARδ and PPARγ, forms a small subfamily of PPARs within the superfamily of nuclear receptors [3]. Similarly to other nuclear receptors, PPARα acts as a heterodimer with the retinoid X receptor and occupies specific DNA sequences referred to as PPAR response elements. Binding of a ligand to PPARα leads to the dissociation of co-repressors and the association of co-activators necessary for the activation of transcription. In mice, PPARα has the highest expression level in liver and brown adipose tissue, followed by a lower expression in kidney, heart, and intestine [4]. The physiological role of PPARα in liver is to govern lipid metabolism. In particular during starvation, activation of PPARα leads to stimulation of fatty acid (FA) uptake, mitochondrial β-oxidation, peroxisomal FA oxidation, and ketogenesis [5, 6]. In addition, PPARα controls lipoprotein metabolism [7–10]. Due to the role of PPARα in maintenance of lipid homeostasis, it has become an important molecular target for dyslipidemia [11]. In addition, PPARα ligands and dual PPARα/PPARδ ligands are being explored for the treatment of non-alcoholic fatty liver disease [12].

Apart from its profound role in regulation of lipid metabolism, PPARα exerts anti-inflammatory and anti-atherogenic effects, which may be beneficial in the treatment of several metabolic diseases associated with inflammation such as non-alcoholic steatohepatitis and atherosclerosis [11, 13]. The anti-inflammatory actions of PPARα are mediated via its negative interference with other nuclear factors such as the NF-κB, AP-1, C/EBP, and STAT proteins, which govern innate and adaptive immunity [14, 15]. In the liver, hepatocytes likely are the major cell type mediating the anti-inflammatory effects of PPARα, since no PPARα was detected in rat liver resident macrophages i.e. Kupffer cells [16, 17]. In addition to hepatocytes, PPARα is expressed in other relevant immune cells such as dendritic cells, non-hepatic macrophages, as well as B and T lymphocytes, which can infiltrate the liver during development of metabolic disorders. Accordingly, these cells are potential therapeutic targets for PPARα ligands to manage hepatic inflammation [18–20]. PPARα is also expressed in non-hepatic vascular cells, likely accounting for the effects of synthetic PPARα agonists on angiogenesis [21, 22], endothelial permeability [23], and vascular adhesion capacity [11], which are important in the treatment of atherosclerosis. However, it is questionable whether PPARα agonists can directly affect hepatic endothelial cell gene expression, because mouse and rat endothelial cells do not express PPARα [16, 17, 24].

Although PPARα is expressed in the liver parenchymal fraction (i.e. hepatocytes) and is not or very weakly expressed in the rodent non-parenchymal liver fraction (i.e. Kupffer cells and endothelial cells) [16, 17, 24], it is presumed that interactions between both fractions as well as inter-organ interactions are necessary to fulfill hepatic PPARα functions related to regulation of lipid metabolism and immunity. In order to determine how cellular composition and inter-organ interactions influence gene expression upon PPARα activation, we performed a meta-analysis of transcriptomics data obtained from relevant mouse liver models represented by mouse primary hepatocytes (containing only the parenchymal fraction) treated with the PPARα agonist Wy14643 for 6 or 24 h (*in vitro*), precision cut liver slices (containing both parenchymal and non-parenchymal fractions) treated with Wy14643 for 24 h (*ex vivo*), and livers of wild type (WT) and Ppara knockout (KO) mice treated with Wy14643 for 6 h or 5 days (*in vivo*). The aim was to obtain a comprehensive view of common and model-specific PPARα-dependent genes and biological processes to understand the impact of the cross-talk between parenchymal and non-parenchymal fractions as well as the effect of inter-organ interactions on the hepatic PPARα transcriptome. In addition, we aimed to assess the performance of *in vitro* and *ex vivo* liver models to study the PPARα transcriptome in relation to the *in vivo* situation.

## Methods

### Chemicals

Wy14643 was obtained from ChemSyn Laboratories (Lenexa, KS). Recombinant human insulin (Actrapid) was obtained from Novo Nordisk (Copenhagen, Denmark). DMEM, fetal calf serum, calf serum, and penicillin/streptomycin/fungizone were obtained from Lonza Bioscience (Verviers, Belgium). Williams E medium supplemented with Glutamax, penicillin/streptomycin (pen/strep), D-glucose, phosphate buffered saline (PBS) were obtained from Invitrogen (Invitrogen, Bleiswijk, The Netherlands). Otherwise, chemicals were obtained from Sigma (Zwijndrecht, The Netherlands).

### Mouse primary hepatocytes (*in vitro*liver model)

Mouse hepatocytes were prepared and used in experiments have been described previously [25]. Briefly, the hepatocytes were isolated by two-step collagenase perfusion from 6 different strains of mouse; NMRI, SV129, FVB, DBA, BALB/C and C57BL/6. The mouse strains differed with respect to age (2–6 months) and gender (female or male) [25]. Cells were plated on collagen-coated six-well plates. Cell viability was determined by Trypan Blue exclusion, and was at least 75%. Hepatocytes obtained for 6 different mouse strains were cultured independently in William’s E medium (Lonza Bioscience, Verviers, Belgium) supplemented with 10% (v/v) fetal calf serum, 20 m-units/mL insulin, 10 nM dexamethasone, 100 U/mL penicillin, 100 mg/mL of streptomycin, 0.25 mg/mL fungizone and 50 mg/mL gentamycin. After four hours the medium was discarded and replaced with fresh medium. The next day, cells were incubated in fresh medium in the presence or absence of Wy14643 (10 μM) dissolved in dimethyl sulfoxide (DMSO) for 6 and 24 h, followed by RNA isolation. Isolation of mouse primary hepatocytes was approved by the Ethical Committee for Animal Experiments of Wageningen University.

### Precision cut liver slices (*ex vivo*liver model)

Precision cut liver slices (PCLS) were prepared and cultured as described previously [26]. Briefly, livers from 6 months old C57BL/6 mice were perfused with saline, excised and submerged in ice-cold Krebs-Henseleit Buffer (KHB) supplemented with 11 mM glucose, 25 mM sodium bicarbonate, 10 mM 4-(2-hydroxyethyl)-1-piperazineethanesulfonic acid (HEPES), and penicillin/streptomycin. The livers were collected at ~11 a.m. (ZT5). Next, 5 mm cylindrical liver cores were prepared with a surgical biopsy punch and sectioned to 200 μm slices using a Krumedieck tissue slicer (Alabama Research and Development, Munford, AL, USA) filled with carbonated KHB. Liver slices were incubated in William’s E Medium (Lonza, Verviers, Belgium) in 6-well plates at 37°C/5% CO_2_/80% O_2_ under continuous shaking. After 1 hour the media was replaced with fresh William’s E Medium in the presence or absence of Wy14643 (20 μM) dissolved in dimethyl sulfoxide (DMSO). After 24 h incubation, samples were snap-frozen in liquid nitrogen and stored in −80°C for RNA isolation. PCLS were obtained from four mice and were cultured independently per each experimental condition i.e. *n* = 4 for Wy14643 and *n* = 4 for DMSO.

### Animals (*in vivo*liver model)

*In vivo* treatment with Wy4643 has been described previously [27]. Briefly, male wildtype and Ppara-KO mice on a Sv129 background were used at 3–5 months of age (Jackson Laboratories, Bar Harbor, ME). Animals were fed normal laboratory chow (RMH-B diet, Arie Blok animal feed, Woerden, the Netherlands). During acute pharmacological activation of PPARα by Wy14643, wildtype and Ppara-KO mice fasted for 4 hours received a single dose of Wy14643 (400 μL of 10 mg/mL Wy14643 dissolved in 0.5% carboxymethylcellulose) and were killed 6 hours later (*n =* 5 per group). During chronic pharmacological activation of PPARα by Wy14643, WT and Ppara-KO mice were fed with Wy14643 for 5 days by mixing it in their food (0.1%, *n =* 5 per group). Livers from the acute pharmacological activation of PPARα were collected at ~3 p.m. (ZT9) and livers from the chronic activation of PPARα were collected at ~11 a.m. (ZT5). Livers from all experiments were dissected and immediately frozen in liquid nitrogen for RNA isolation. All animal experiments were approved by the Ethical Committee for Animal Experiments of Wageningen University.

### RNA isolation

Total RNA was extracted from primary hepatocytes using TRIzol reagent (Invitrogen, Breda, The Netherlands) and RNA was further purified using RNeasy micro columns (Qiagen, Venlo, the Netherlands).

Prior to RNA isolation, PCLS were homogenized with a tissue homogenizer Precellys 24 Bertin Technologies (Labmakelaar Benelux B.V. Rotterdam, The Netherlands) using settings: 2x (15 sec, 6500 *g,* 8°C). Next, total RNA was isolated using the RNeasy Tissue Mini Kit (Qiagen, Venlo, The Netherlands) according to manufacturer’s protocols.

Total RNA was extracted from mouse livers using TRIzol reagent (Invitrogen, Breda, The Netherlands), and purified and DNAse treated using the SV Total RNA Isolation System (Promega, Leiden, The Netherlands).

RNA concentration and purity were assessed spectrometrically using a Nano Drop ND-1000 spectrophotometer (Isogen, IJsselstein, The Netherlands). RNA quality was assessed on an Agilent 2100 bioanalyzer (Agilent Technologies, Amsterdam, the Netherlands). Only RNA samples that met quality criteria (i.e. RNA Integrity Number >8.0) were used for array hybridization experiments. For primary hepatocytes, purified RNA (500 ng) was used for one cycle cRNA synthesis (Affymetrix, Santa Clara, CA). Hybridization of samples derived from primary hepatocytes was performed on Affymetrix Gene chip mouse genome 430 2.0 arrays. Purified RNA (100 ng) isolated from PCLS was labeled with the Ambion WT expression kit (Life Technologies, Bleiswijk, The Netherlands) and hybridized to Affymetrix Mouse Gene 1.1 ST array plate (Affymetrix, Santa Clara, CA). RNA isolated from mouse livers (5 μg) was used for one cycle cRNA synthesis (Affymetrix, Santa Clara, CA) and hybridized to Affymetrix Genechip mouse genome 430 2.0 arrays (wildtype and Ppara-KO treated with Wy14643 or vehicle for 6 hours as well as wildtype and Ppara-KO treated with Wy14643 or vehicle for 5 days). Hybridization, washing, and scanning of all Affymetrix Genechips was according to standard Affymetrix protocols. Scans of the Affymetrix arrays were processed using the Bioconductor package [28]. The microarray data obtained from the above mentioned experiments were deposited to gene expression omnibus (GEO). The GEO series accession numbers are as follows: GSE17250 (primary hepatocytes obtained from 6 different mouse strains treated with Wy14643 or vehicle, n = 6 per group), GSE8292 (WT and Ppara-KO treated with Wy14643 or vehicle for 6 hours, n = 3-5 per group), and GSE8295 (WT and Ppara-KO treated with Wy14643 or vehicle for 5 days, n = 4 per group). The accession number for the microarray of precision cut liver slices is pending.

### Statistical analysis of microarray data

Expression levels of probe sets were calculated using Robust Multiarray Averaging (RMA) with m-estimator summarization and remapped in Common Data Format (CDF) version 17.0.1 [29]. Identification of differentially expressed genes was performed using Rank Products (RP). False discovery rates were determined by RP using 1000 permutations of the samples. Genes with a false discovery rate (FDR) ≤ 0.05 were considered as significant [30]. All comparisons were made between treatments and controls (i.e. Wy14643 vs. vehicle).

### Gene ontology analysis -DAVID

The significantly up- and down-regulated genes by the treatments (i.e. Wy14643) were uploaded separately to the Database for Annotation, Visualization, and Integrated Discovery (DAVID) Bioinformatics Resource. In DAVID the Functional Annotation Clustering tool generated clusters of up- or down- overrepresented Gene Ontology (GO) terms [31, 32]. The Mouse Genome 430 2.0 was used as a background for the GO analysis of the *in vitro*, *ex vivo*, and *in vivo* hepatic models. After correction for FDR ≤ 0.005 (Benjamini Hochberg), the GO terms were selected for further analysis and interpretation.

### Venn diagram analysis

Venn diagram analysis of the significantly affected genes identified in our study was performed using VENNY [33].

### Pearson’s correlation analysis

Pearson’s correlation analysis was performed using IBM SPSS Statistics 20 and the results are presented as scatterplots using Microsoft Excel 2010.

## Results

### Identification of differentially expressed genes

**Figure 1:**
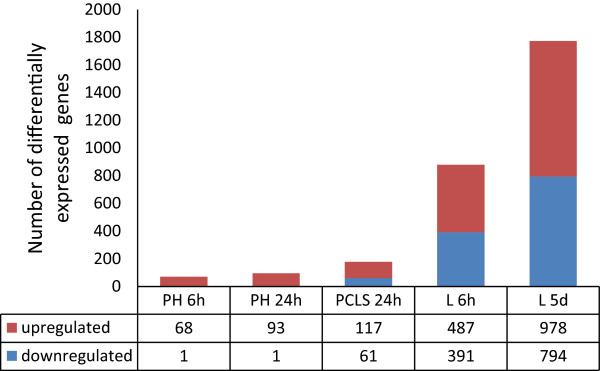
**Effect on hepatic gene expression during activation of PPARα.** The total number of significantly up- and down-regulated genes (FDR ≤ 0.05) identified in primary hepatocytes treated with Wy14643 for 6 or 24 h (PH 6 h or PH 24 h), precision cut liver slices treated with Wy14643 for 24 h (PCLS 24 h), livers of mice treated with Wy14643 for 6 h of 5 days (L6h or L5d).

In order to identify genes significantly altered by pharmacological PPARα activation, we analyzed microarray data of different liver models (i.e. *in vitro*, *ex vivo*, and *in vivo*) treated with the synthetic PPARα agonist Wy14643 using Rank Products (RP) [30]. The use of Wy14643 was justified by the observation that changes in *in vivo* hepatic gene expression elicited by Wy14643 and another common PPARα agonist fenofibrate were extremely similar (Additional file 1). Only genes that were overlapping between different types of microarray chips used in our study were included in the analysis. In general it was observed that the number of significantly altered genes increased with increasing complexity of the liver model (with regard to duration of the treatment and cellular composition of the model) (Figure 1). Thus, in primary hepatocytes treated with Wy14643 for 6 h and 24 h, 68 and 93 genes were upregulated, respectively. In liver slices, 117 genes were significantly upregulated by Wy14643, and in livers of mice exposed to Wy14643 for 6 h and 5 days, 487 and 978 genes were significantly upregulated, respectively. Very few genes were downregulated by PPARα activation in primary hepatocytes, whereas 61, 391 and 794 genes were significantly downregulated by Wy14643 treatment in liver slices, mouse livers treated for 6 h, and mouse livers treated for 5 days, respectively.

### Relevance of *in vitro*and *ex vivo*models for the *in vivo*situation

**Figure 2:**
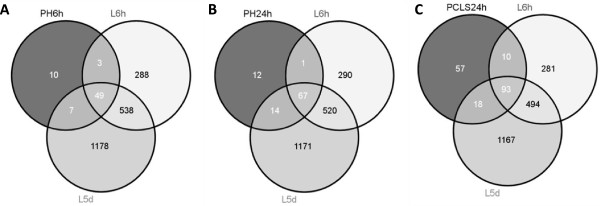
**Venn diagram analysis.** Venn diagram analysis of the significant genes (FDR ≤ 0.05) identified in “simpler” models represented by **(A)** primary hepatocytes treated with Wy14643 for 6 h (PH 6 h), **(B)** primary hepatocytes treated with Wy14643 for 24 h (PH 24 h), and **(C)** precision cut liver slices treated with Wy14643 for 24 h (PCLS 24 h) vs. significant genes (rank products, FDR ≤ 0.05) identified in *in vivo* models represented by livers of mice exposed to Wy14643 for 6 h (L6h) and livers of mice exposed to Wy14643 for 5 days (L5d).

To determine the resemblance between “simpler” liver models (i.e. *in vitro* and *ex vivo*) vs. the more complex models (i.e. *in vivo*), Venn diagram analysis of genes significantly regulated in each model was performed. The analysis showed that there was a substantial overlap between the “simpler” hepatic models and the *in vivo* models, Figure 2A-C.

**Figure 3:**
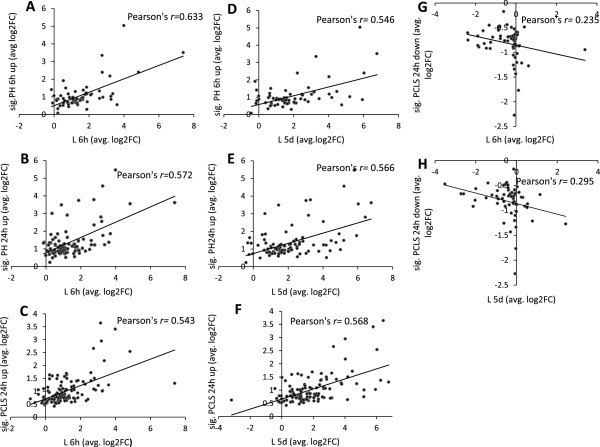
**Pearson’s correlation analysis.** Significantly (sig.) upregulated genes (FDR ≤ 0.05) identified in primary hepatocytes (PH) treated with Wy14643 for 6 or 24 h and precision cut liver slices (PCLS) treated with Wy14643 for 24 h were correlated with corresponding genes in livers (L) of mice treated with Wy14643 for 6 h **(A-C)** or 5 days (5d), **D-F)**. Significantly downregulated genes (FDR ≤ 0.05) identified in PCLS 24 h were correlated with corresponding genes in L6 h **(G)** or L5d **(H)**. Each dot represents the average (avg.) log2 value of fold change (FC) in gene expression of treatment group vs. control. All correlations were significant at P ≤ 0.01, with the exception of 3G and 3H.

Additionally, to evaluate whether Wy14643 affected gene expression in the same direction in *in vitro*/*ex vivo* versus *in vivo* models, and to compare the magnitude of fold changes in each model, Pearson’s correlation analysis was performed (Figure 3). In the analysis we separately compared expression changes of genes significantly upregulated (FDR ≤ 0.05) in primary hepatocytes and liver slices after Wy14643 treatment with expression changes of the corresponding genes *in vivo*. Interestingly, we observed that the majority of the genes analyzed were commonly upregulated between *in vitro/ex vivo* and *in vivo* experiments. The magnitude of gene expression changes was generally higher *in vivo* compared to *in vitro* and *ex vivo* (Figure 3A-F).

The same type of analysis was performed for the significantly downregulated genes (FDR ≤ 0.05) identified *ex vivo*, i.e. expression of genes significantly downregulated in liver slices was compared with expression changes of the corresponding genes *in vivo*. Surprisingly, out of 61 significantly downregulated genes in liver slices, only 23 and 31 corresponding genes were changed in the same direction in livers of mice treated with Wy14643 for 6 h and 5 days, respectively, while the remaining genes were unaltered or upregulated *in vivo* compared to *ex vivo*. In general, fold changes of the commonly downregulated genes were lower in liver slices compared to livers *in vivo* (Figure 3G-H). Due to the absence of significantly downregulated genes in primary hepatocytes, no correlation analysis was performed for this model.

### Gene ontology analysis

**Figure 4:**
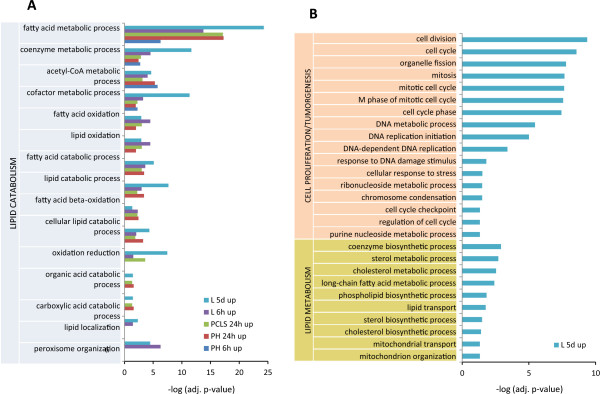
**Gene Ontology (GO) analysis of significantly upregulated genes in different hepatic models.** Significantly (FDR ≤ 0.005) enriched GO terms related to lipid catabolism were identified in most of the liver models studied, **A**. GO terms related to cell proliferation/tumorgenicity and other aspects of lipid metabolism were significantly enriched (FDR ≤ 0.005) in livers of mice treated with Wy14643 for 5 days (L5d), **B**. PH stands for primary hepatocytes, PCLS-precision cut liver slices, and L-liver.

In order to obtain a comprehensive view of the biological processes affected by Wy14643 in different liver models, significantly up- and down-regulated genes were subjected to GO analysis using DAVID [32, 34]. The GO analysis of the upregulated genes by Wy14643 in all liver models revealed that several GO terms related to different aspects of lipid catabolism such as “fatty acid metabolic process” and “acyl-CoA metabolic process” were significantly enriched in almost all hepatic models, except for “lipid localization” and “peroxisomes organization”, which were found only *in vi*vo (Figure 4A). However, GO terms related to cell proliferation/tumorgenesis and other aspects of lipid metabolism were significantly enriched only in livers of mice exposed to Wy14643 for 5 days (Figure 4B).

**Figure 5:**
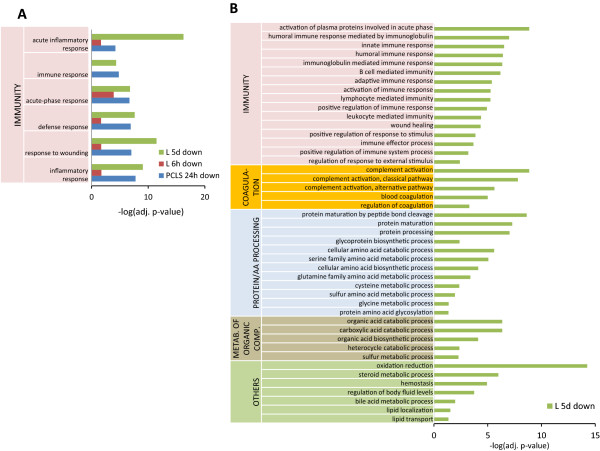
**Gene Ontology (GO) analysis of significantly downregulated genes in different hepatic models.** Significantly (FDR ≤ 0.005) enriched GO terms related to different aspects of immunity were identified in liver slices treated with Wy14643 for 24 h (PCLS 24 h) and livers of mice treated with Wy14643 for 6 h (L6h) or 5 days (L5d), **A**. GO terms related to other aspects of immunity, coagulation, protein/amino acid (aa) metabolism, metabolism of organic compounds, and others were significantly (FDR ≤ 0.005) enriched only in livers of mice treated with Wy14643 for 5 days (L5d), **B**.

The GO analysis performed for the significantly downregulated genes showed that GO terms related to different aspects of immunity were significantly enriched in both *ex vivo* and *in vivo* models (Figure 5A). GO terms exclusively detected in livers of mice exposed to Wy14643 for 5 days were related to immunity, coagulation, protein/amino acid metabolism, metabolism of organic compounds as well as GO processes nominated as “oxidation reduction”. “steroid metabolic process”, “hemostasis”, “regulation of body fluid levels”, “bile acid metabolic process”, “lipid localization”, and “lipid transport” (Figure 5B).

### “PPARα-tailored analysis”

**Figure 6:**
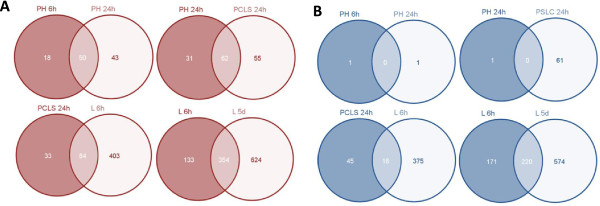
**Gene selection for the “PPARα tailored analysis”.** Genes selected for the “PPARα tailored analysis” consisted of significantly up- and down-regulated genes (FDR ≤ 0.05) that were overlapping between at least two liver models. Numbers of overlapping up- and down-regulated genes are presented in **A** and **B** respectively. PH 6 h/24 h stands for primary hepatocytes treated with Wy14643 for 6 or 24 h, PCLS 24 h-precision cut liver slices treated with Wy14643 for 24 h, and L6 h/5d-liver treated with Wy14643 for 6 h of 5 days.

Due to the fact that the GO analysis resulted in functional annotation of only a sub-fraction of the total number of significant genes (~25% genes per liver model), an additional functional analysis, referred to as “PPARα-tailored analysis”, was performed. The incentive for this additional analysis was to annotate a higher number of genes than via GO analysis and assign genes to novel and more detailed functional categories. The “PPARα-tailored analysis” was performed gene by gene using the open access database GeneCards. GeneCards provides current genomic, proteomic, genetic, transcriptomic, and functional information on all known and predicted genes. The selection of genes for the “PPARα-tailored analysis” was rather subjective; we considered all significant genes that were overlapping between at least two hepatic models (Figure 6A-B). Eventually we considered 370 unique upregulated genes (out of 1120 unique genes upregulated in all hepatic models) and 224 unique downregulated genes (out of 991 unique genes downregulated in all hepatic models). The aim behind such a selection was to focus on the most relevant genes for PPARα signaling across all liver models and at the same time reduce the total number of genes analyzed. We only analyzed genes that are regulated in a PPARα-dependent manner. Thus genes that were significantly regulated by Wy14643 treatment in both wildtype and Ppara-KO were excluded from the “PPARα-tailored analysis”. Using GeneCards we were able to functionally annotate 227 (out of 370 upregulated) and 125 (out of 224 downregulated) genes, which were divided into up- and down-regulated functional categories (Additional files 2, 3 and 4). As part of the “PPARα-tailored analysis”, genes related to lipid catabolism were categorized into “peroxisomal β-oxidation”, “peroxisomal membrane & biogenesis”, and “mitochondrial carriers” and these categories, similarly to the results from GO analysis, contained significantly upregulated genes in all liver models (Additional file 2). However, the more complex the hepatic model, the higher the number of significant genes and their fold change. As in GO analysis, a gene functional category related to tumorgenesis (i.e. cell cycle) contained genes that were significantly upregulated only in *in vivo* models (Additional file 3).

In comparison to GO analysis, the “PPARα-tailored analysis” led to the identification of additional upregulated functional categories such as “extracellular matrix” (ECM), “cytoskeleton”, “endothelial functions”, “integrity of Golgi”, “vesicular trafficking”, “endocytosis”, and “neurotransmission”. Genes belonging to these categories were significantly upregulated almost exclusively *in vivo* (Additional files 2 and 3). Downregulated functional categories such as “acute phase” or “coagulation” contained genes that were significantly downregulated mostly *in vivo*; only few genes within these categories were also significantly downregulated in liver slices (e.g. F11, Orm2, Orm3, and Crp) (Additional file 3). In addition, compared to GO analysis, the “PPARα-tailored analysis” identified novel downregulated gene functional categories such as “transcriptional co-repression/activation”, “NF-κB (downstream activation)”, “immunity”, “T and B cells functions”, “angiogenesis”, “ECM”, “cytoskeleton”, and “neurotransmission”. Genes belonging to these categories were significantly and nearly exclusively downregulated *in vivo*, with the exception of a few genes that were also significantly downregulated *ex vivo* and none of these genes were significantly altered *in vitro* (Additional file 4).

**Figure 7:**
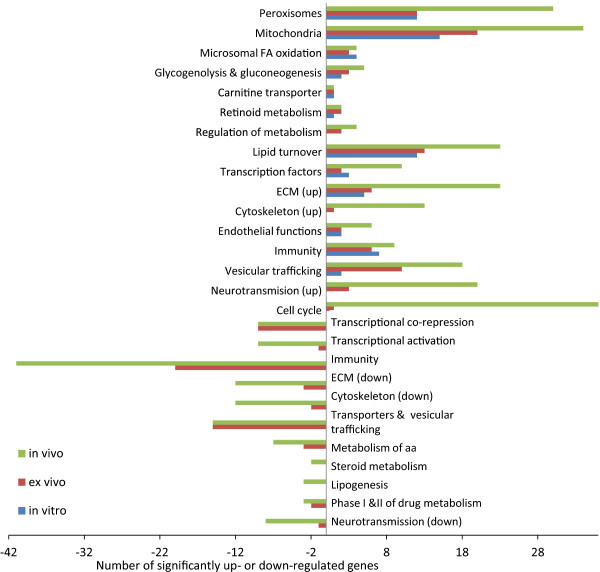
**Summary of the “PPARα tailored analysis”.** Genes selected for the “PPARα-tailored analysis” were annotated using GeneCards and grouped into functional categories such as “peroxisomes”, “mitochondria” etc. The total number of significantly up- and down-regulated genes found per category in *in vitro*, *ex vivo*, and *in vivo* models is represented by horizontal bars. Bars located >0 on Y axis show number of upregulated genes and bars located <0 on Y axis show number of downregulated genes. *In vitro* represents primary hepatocytes treated with Wy14643 for 24 h, *ex vivo* - precision cut liver slices treated with Wy14643 for 24 h and *in vivo* - livers of mice treated with Wy14643 for 5 days.

In general, the results of the “PPARα-tailored analysis” were in line with the statistical and GO analyses and revealed that the most significant changes occurred *in vivo*, followed by *ex vivo*, and the least changes occurred in *in vitro* models. However, compared to GO analysis, the “PPARα-tailored analysis” annotated a higher percentage of the analyzed genes (i.e. ~50% vs. ~25% respectively) as well as identified additional and novel PPARα-related functional categories. A summary of the results of the ‘PPARα- tailored analysis’ is presented in Figure 7.

### Integrated concept of PPARα signaling during its pharmacological activation

**Figure 8:**
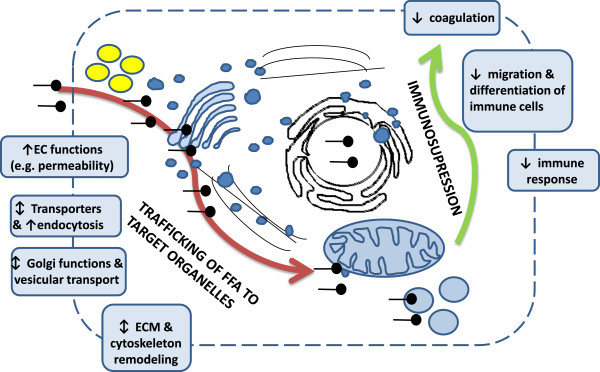
**An integrated picture of PPARα biology.** Gene functional categories identified in the “PPARα tailored analysis” were integrated into a simplified concept of hepatic PPARα biology. The picture represents a hepatocyte and organelles such as lipid droplets (yellow spheres), Golgi, transport vesicles (irregular blue spheres), nucleus, mitochondria, peroxisomes (regular light blue spheres), and trafficking FFA (black figures symbolizing hydrophobic and hydrophilic parts of FFA). Text boxes contain the identified functional categories and arrows indicate direction of changes of the significant genes (FDR ≤ 0.05) within the gene functional categories.

Biological processes identified in the meta-analysis were integrated into a simplified concept of hepatic PPARα signaling under not-inflamed conditions (Figure 8). We propose that during activation of PPARα, significant regulation of genes related to “ECM” and “cytoskeleton” as well as “endothelial functions”, “endocytosis”, “Golgi”, and “vesicular trafficking” might facilitate sudden influx and traffic of FA to hepatocytes and target organelles such as nucleus, mitochondria, and peroxisomes. At the same time, activation of PPARα leads to immunosuppression caused by downregulation of genes related to “NF-κB signaling”, “coagulation”, “acute phase”, and “immunity”, likely to suppress an immune response elicited by massive traffic of potentially cytotoxic FA [35]. Finally, pharmacological activation of PPARα affects genes involved in “neurotransmission”.

## Discussion

The challenge in meta-analyses is to circumvent the variation of independent microarray experiments and extract biologically relevant information [36]. The meta-analysis of hepatic PPARα signaling performed in our study is mostly based on separate microarray experiments that were generated over several years and originally served a different purpose than this study [25, 27]. Variations in the experimental set up used to generate these microarray data are: use of different types of Affymetrix gene chips, different batches of chemicals, unequal number of biological replicates per group, differences in time of exposure to Wy14643 and model-specific concentration of Wy14643. Furthermore, there were differences related to age, gender, and mouse strains, which all together could increase experimental variation and hamper the identification of genuine biological effects. However, the experiments were performed on the same platform, by the same technician, and standard procedures were used over the years.

To minimize the effects of the above-mentioned experimental differences, and to allow identification of biologically relevant significant genes, we applied the RP approach. The RP approach is a powerful statistical method to analyze microarray data generated from relatively few replicates, performed in different laboratories and/or using different platforms. Moreover, RP outperformed other statistical tests applied in meta-analyses such as t-based approach and Fisher’s Inverse χ^2^ method [30, 36, 37].

In our study, we identified a variable number of significantly up- and down-regulated genes in different liver models. The number of significantly regulated genes corresponded with the complexity of the hepatic model determined by time of exposure, cellular composition, and the absence/presence of systemic effects (i.e. inter-organ interactions). Thus, the lowest number of significantly upregulated genes was found in primary hepatocytes and in this model very few genes were significantly downregulated. This finding contradicts with our previous analysis in which we identified more than 400 up- and down-regulated genes in primary hepatocytes after 6 and 24 h treatment with Wy14643 [25]. This discrepancy can be explained by the application of different statistical methods in both studies, i.e. the former study used a Linear Models for Microarray Data without correction for FDR. In contrast, we applied a more stringent RP that corrects for FDR [25, 28, 30].

The very low number of downregulated genes in primary hepatocytes may be explained by the absence of the non-parenchymal fraction, which is an abundant source of pro-inflammatory factors [38]. Although it was shown that activation of PPARα can suppress the immune response and downregulate gene expression in hepatocytes by antagonizing the NF-κB signaling pathway, the relevant experiments were performed in the presence of pro-inflammatory stimuli [14]. In contrast, our experiments in hepatocytes were performed under non-inflamed conditions, which may explain the very low number of significantly downregulated genes. In agreement with our results, a study performed in rat liver and rat primary hepatocytes treated with PPARα ligands showed that downregulation of (proinflammatory) genes occurred only *in vivo* [39].

In order to evaluate the relevance of *in vitro* and *ex vivo* models for the *in vivo* situation, we performed a comparative analysis of the significantly altered genes in each model by means of Venn diagrams. In addition, Pearson’s correlation analysis was performed to compare the direction of changes in gene expression in *in vitro* and *ex vivo* models vs. the *in vivo* situation. The results of Venn diagram analysis showed substantial similarities between “simpler” liver models and the *in vivo* models, suggesting that primary hepatocytes and liver slices are relevant models to study pharmacological activation of PPARα *in vivo*. With regard to the results of Pearson’s correlation analysis, it revealed that almost all genes upregulated *in vitro* and *ex vivo*, were also upregulated *in vivo*. In contrast, only a small fraction of genes downregulated *ex vivo* was downregulated *in vivo*. These results indicate that primary hepatocytes and liver slices are valid liver models to study genes induced by activation of PPARα with Wy14643, but less suitable to study genes downregulated by activation of PPARα with Wy14643. This finding was unexpected and suggests that downregulation of genes by PPARα is model-dependent. Inasmuch as the cellular composition of liver slices is comparable to the liver *in vivo*, i.e. both models contain parenchymal and non-parenchymal cells [40], these differences may be explained by systemic effects that are obviously absent *ex vivo*.

In all liver models, lipid metabolism emerged as a dominant pathway regulated by PPARα [25, 27]. Despite the fact that there is a cross-talk between parenchymal and non-parenchymal fractions in regulation of hepatic lipid metabolism [41–43], we did not observe striking differences in regulation of genes involved in lipid metabolism between our *in vitro* and *ex vivo* models, suggesting that the presence of non-parenchymal cells does not affect the Wy14643-mediated regulation of genes involved in lipid metabolism. Genes related to lipid metabolism were induced most significantly (in terms of number and fold change) *in vivo*, indicating that lipid metabolism is more sensitive to regulation *in vivo* compared to *in vitro* and *ex vivo* models. However, it can’t be ruled out that our results are biased by the lack of standardization of conditions across all liver models in relation to differences in concentration of glucose, insulin, or other biomolecules, which could also affect lipid metabolism.

Next to the identification of processes related to different aspects of lipid catabolism, the “PPARα-tailored analysis” identified genes, mostly *in vivo*, that could be assigned into novel functional categories not previously linked to PPARα, such as “ECM”, “cytoskeleton”, “endothelial functions”, “integrity of Golgi”, “vesicular trafficking”, and “endocytosis”. Given the key role of PPARα in regulation of hepatic lipid metabolism, we hypothesize that genes belonging to these novel categories might somehow be linked to transport, traffic, and catabolism of FA during activation of PPARα by Wy14643. It is possible that during pharmacological activation of PPARα, FA and TG carried by lipoproteins and albumin freely pass fenestrated hepatic endothelial cells [27, 44]. In hepatocytes, FA are taken up by transporters [45], followed by poorly defined trafficking of FA to target organelles such as mitochondria and peroxisomes to undergo oxidation. Consistent with this picture, we hypothesize that genes categorized as “endothelial functions” and “endocytosis” could be involved in transfer of FA through the endothelium, exemplified by the known PPARα target Angptl4, which plays a role in vascular permeability [46]. In addition, Adtrp, Nrpt, E2f8 could also modulate endothelial permeability via interaction with Vegf-b [47]. Next, the traffic of FA to target organelles might be governed by genes in categories denoted as “ECM”, “cytoskeleton”, “integrity of Golgi”, “endocytosis”, and “vesicular trafficking”. This notion is in line with an emerging concept of vesicular transport of FA to target organelles and cells co-occurring with reorganization of ECM, cytoskeleton, and Golgi [48]. Interestingly, similar functional categories have been linked to PPARβ/δ in pancreatic β–cells including granule biosynthesis, intra-cellular vesicle trafficking, and exocytosis [49]. Therefore, it could be hypothesized that all PPARs may play a role in traffic of certain molecules in and out of the cell.

In addition to the above mentioned processes, several GO processes and genes related to different aspects of immunity were significantly downregulated only in *ex vivo* and *in vivo* models (Figure 5, Additional file 3). These findings are in concordance with the known anti-inflammatory properties of PPARα [11, 14]. However, in our study, due to the lack of inflammation, it seems to be more appropriate to refer to these processes as immunosuppression. Immunosuppression was not observed in primary hepatocytes, suggesting that interaction with immune mediators and/or immune cells (e.g. Kupffer cells) is essential.

Another novel gene functional category identified in this study exclusively *in vivo* is denoted as “neurotransmission”. Currently, there is a scarce evidence for a crosstalk between PPARα and the nervous system. However, recently it was shown that stimulation of the vagus nerve increased plasma endogenous PPARα ligands co-occurring with upregulation of hepatic PPARα expression and systemic anorectic effects [50]. Another study showed that hepatic PPARα activation led to glucocorticoid-induced insulin resistance and hypertension via an afferent vagal nerve pathway [51]. It is possible, therefore, that activation of PPARα by Wy14643 affects neurosignaling to coordinate liver metabolism with other organs and tissues to maintain energy homeostasis.

Finally, in accordance with the known hepatocarcinogenic effect of peroxisomes proliferators in rodents [52], we found that activation of PPARα significantly upregulated several genes related to cell proliferation *in vivo*. Surprisingly, this effect was absent in our *in vitro* and *ex vivo* liver models. Given the fact that Wy14643 induces DNA synthesis in rat hepatocytes after 48 hours, but not 24 hours [53], it can be speculated that the 24 hours Wy14643 treatment in our *in vitro* model might be too short to detect the proliferative/carcinogenic changes. Previously it was reported that treatment of mouse primary hepatocytes with peroxisomes proliferators (including Wy14643) for 24 hours led to upregulation of 11 genes related to liver cancer (i.e. Angptl4, Bnip3, Dbi, Fabp1, Fabp2, Fasn, HifIa, Lgals3, Ly6d, Mgll, SerpineI) [54]. However, it has to be stressed that none of these genes is strictly related to cell proliferation/carcinogenesis [GeneCards]. Consistent with the notion that Kupffer cells play a central role in peroxisome proliferator-induced carcinogenesis, it was found that genes related to cell proliferation are induced by PPARα ligands in rat liver but not in primary rat hepatocytes [39].

Our observation that treatment of liver slices with Wy14643 did not alter genes related to cell proliferation conflicts with the finding that peroxisomes proliferators induced DNA synthesis in rodent co-cultures of hepatocytes with non-parenchymal cells [41, 53]. A possible explanation for this discrepancy might be that to assess proliferative/carcinogenic properties of peroxisomes proliferators, measuring DNA synthesis is more appropriate than analysis of genes related to cell proliferation. Unfortunately, we cannot make a comparison of our gene expression data obtained in liver slices with gene expression obtained in other relevant rodent liver models, due to the lack of such data.

## Conclusions

In summary, this study provides a comprehensive picture of gene expression during pharmacological activation of PPARα by Wy14643 in different mouse hepatic models under not-inflamed conditions. PPARα-dependent regulation of many genes and processes involved in lipid metabolism is mostly independent of the presence of non-parenchymal cells or inter-organ interactions, as it was observed in all liver models. PPARα-dependent regulation of inflammatory genes requires the presence of non-parenchymal cells, because it was observed only *ex vivo* and *in vivo*. However, the full spectrum of PPARα biology at the level of lipid metabolism, immunity, carcinogenesis, as well as novel aspects of PPARα signaling such as coagulation, vesicular trafficking and the extracellular matrix, seems to require systemic factors, as it was observed exclusively *in vivo*.

Although *in vitro* and *ex vivo* systems turned out to be relevant liver models to study the PPARα transcriptome compared to the *in vivo* situation, liver slices behaved as an intermediate model between *in vitro* and *in vivo*, and thus represent a superior replacement for primary hepatocytes.

## Electronic supplementary material

Additional file 1: **A comparative analysis of gene expression by Wy14643 and fenofibrate in mouse liver.** Global gene expression data obtained from samples representing livers of mice treated for 6 h with Wy14643 or fenofibrate were expressed as fold change calculated as ratio of gene expression in treatment group (n=4 or 5) vs. gene expression in control group (n=4). (PDF 624 KB)

Additional file 2: **PPARα tailored gene functional analysis (upregulation).** Significantly upregulated genes that were overlapping between at least two hepatic models were selected for a functional analysis using an open access database GeneCards. Based on the provided information, the selected genes were grouped into functional categories and analyzed in each hepatic model i.e. primary hepatocytes treated with Wy14643 for 6 h or 24 h (PH 6H or PH 24 h), precision cut liver slices treated with Wy14643 for 24 h (PCLS 24 h), and livers of mice treated with Wy14643 for 6h or 5 days (L6h or L5d). Genes that were significantly altered are depicted in bold and underlined, red color indicates upregulation, black no change, and green downregulation of gene expression. Fold change was calculated as the average gene expression value in treatment group vs. control. (PDF 522 KB)

Additional file 3: **PPARα tailored gene functional analysis (upregulation).** Significantly upregulated genes that were overlapping between at least two hepatic models were selected for a functional analysis using an open access database GeneCards. Based on the provided information, the selected genes were grouped into functional categories and analyzed in each hepatic model i.e. primary hepatocytes treated with Wy14643 for 6h or 24 h (PH 6H or PH 24 h), precision cut liver slices treated with Wy14643 for 24 h (PCLS 24 h), and livers of mice treated with Wy14643 for 6 h or 5 days (L6h or L5d). Genes that were significantly altered are depicted in bold and underlined, red color indicates upregulation, black no change, and green downregulation of gene expression. Fold change was calculated as the average gene expression value in treatment group vs. control. (PDF 391 KB)

Additional file 4: **PPARα tailored gene functional analysis (downregulation).** Significantly downregulated genes that were overlapping between at least two hepatic models were selected for a functional analysis using an open access database GeneCards. Based on the provided information, the selected genes were grouped into functional categories and analyzed in each hepatic model i.e. primary hepatocytes treated with Wy14643 for 6 h or 24 h (PH 6H or PH 24 h), precision cut liver slices treated with Wy14643 for 24h (PCLS 24 h), livers of mice treated with Wy14643 for 6 h or 5 days (L6h or L5d). Genes that were significantly altered are in bold and underlined, red color indicates upregulation, black no change, and green downregulation. Fold change was calculated as the average gene expression value in treatment group vs. control. (PDF 430 KB)

## Abbreviations

PPARα: Peroxisome proliferator-activated receptor alpha
FA: Fatty acids
PBS: Phosphate buffered saline
DMSO: Dimethyl sulfoxide
KHB: Krebs-Henseleit Buffer
HEPES: (4-(2-hydroxyethyl)-1-piperazineethanesulfonic acid)
PCLS: Precision cut liver slices
KO: Knockout
WT: Wild type
GO: Gene Ontology
DAVID: The Database for Annotation, Visualization, and Integrated Discovery
RP: Rank products
FDR: False discovery rate
RMA: Robust Multiarray Averaging
CDF: Common Data Format
ECM: Extracellular matrix.
