# Supplementary figures and images for: Transcriptomic signatures of peroxisome proliferator-activated receptor α (PPARα) in different mouse liver models identify novel aspects of its biology
Source: BMC Genomics. 2014 Dec 15;15(1):1106. doi: 10.1186/1471-2164-15-1106 (PMC4378209; doi:10.1186/1471-2164-15-1106)

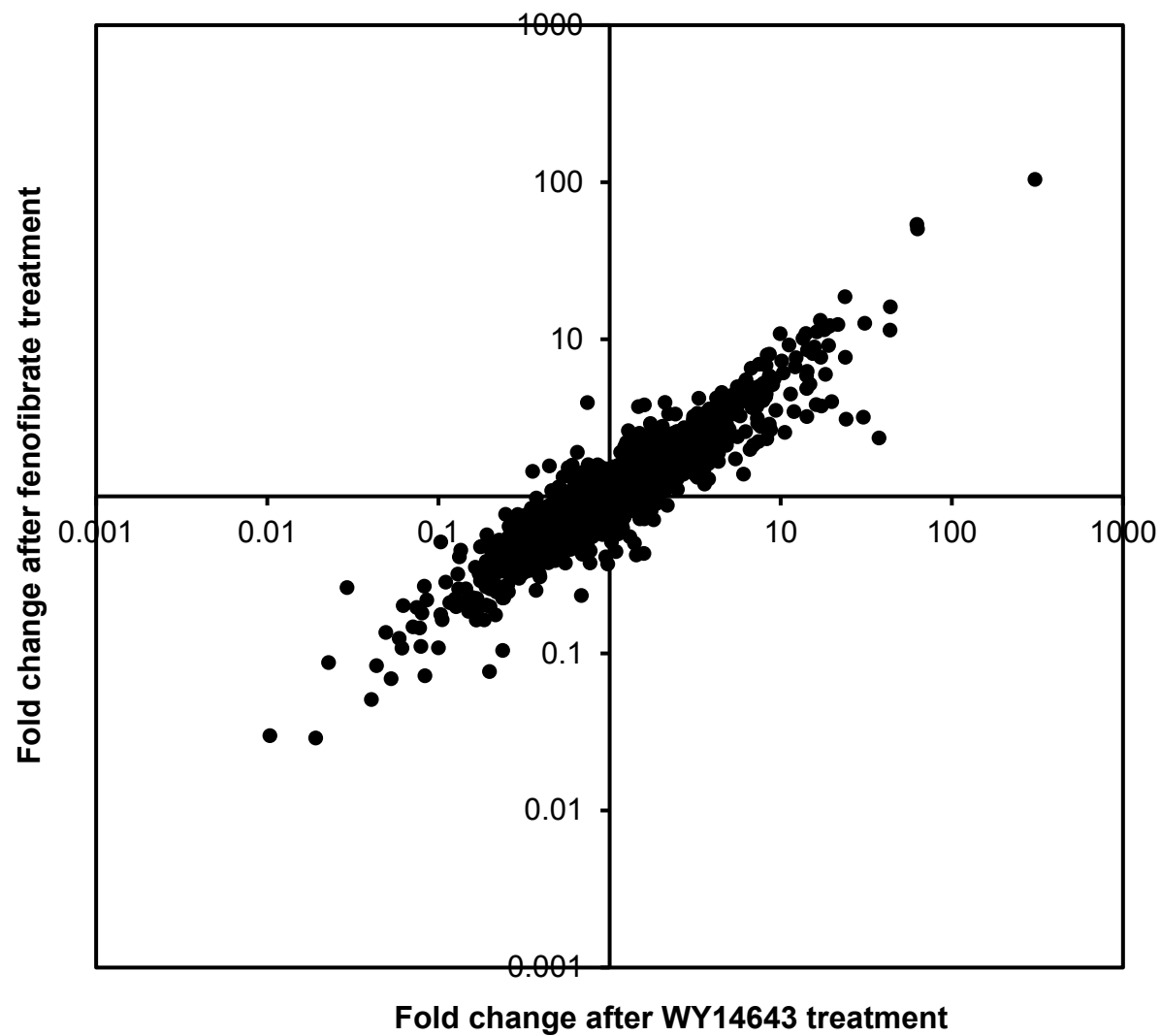

Supplement: Supplementary file 1 — Additional file 1: A comparative analysis of gene expression by Wy14643 and fenofibrate in mouse liver. Global gene expression data obtained from samples representing livers of mice treated for 6 h with Wy14643 or fenofibrate were expressed as fold change calculated as ratio of gene expression in treatment group (n=4 or 5) vs. gene expression in control group (n=4). (PDF 624 KB) [file 12864_2014_6870_MOESM1_ESM.pdf]
