# Supplementary material for: Transcriptomic signatures of peroxisome proliferator-activated receptor α (PPARα) in different mouse liver models identify novel aspects of its biology
Source: BMC Genomics. 2014 Dec 15;15(1):1106. doi: 10.1186/1471-2164-15-1106 (PMC4378209; doi:10.1186/1471-2164-15-1106)
Supplement: Supplementary file 2 — Additional file 2: PPARα tailored gene functional analysis (upregulation). Significantly upregulated genes that were overlapping between at least two hepatic models were selected for a functional analysis using an open access database GeneCards. Based on the provided information, the selected genes were grouped into functional categories and analyzed in each hepatic model i.e. primary hepatocytes treated with Wy14643 for 6 h or 24 h (PH 6H or PH 24 h), precision cut liver slices treated with Wy14643 for 24 h (PCLS 24 h), and livers of mice treated with Wy14643 for 6h or 5 days (L6h or L5d). Genes that were significantly altered are depicted in bold and underlined, red color indicates upregulation, black no change, and green downregulation of gene expression. Fold change was calculated as the average gene expression value in treatment group vs. control. (PDF 522 KB) [file 12864_2014_6870_MOESM2_ESM.pdf]

Additional file 2: PPARα tailored gene functional analysis (upregulation).

Biological function	Genes			PH6h			PH 24h			PCLS 24h			L 6h			L 5d		
Peroxisomal β-oxidation	Ehhadh	Acot4	Acaa1b	10.2	2.3	2.0	13.7	2.3	11.2	6.3	4.5	2.3	6.7	10.3	1.8	9.7	16.4	2.7
	Crot	Acot3	Hsd17b4	1.6	5.3	1.2	1.7	11.7	1.7	2.1	5.8	1.5	1.7	28.5	1.7	2.8	65.4	2.4
	Acox1	Ech1	Hacl1	1.3	1.3	1.0	1.8	2.7	1.4	1.6	1.6	1.7	1.8	1.7	2.2	3.0	5.0	2.3
	Acot12	Acot5	Acot8	1.4	1.3	1.1	1.9	1.2	1.3	1.6	1.3	1.1	1.8	3.4	2.8	2.6	5.4	4.6
	Acaa1a	Decr2	Mlycd	1.1	1.1	1.4	1.4	1.3	1.4	1.3	1.4	1.6	2.2	1.8	1.5	4.7	3.7	2.4
Peroxisomal membrane & biogenesis	Pxmp4	Pex11a	Abcd3	1.8	1.9	1.5	2.0	2.3	1.7	3.0	2.7	1.7	2.1	6.1	2.3	2.4	11.0	3.1
	Pex1	Pex19	Pex13	1.1	1.1	1.0	1.2	1.2	1.1	1.5	1.4	1.2	3.1	2.2	2.0	2.5	2.9	1.7
	Pex14	Pex3	Pex16	1.1	1.0	1.1	1.1	1.1	1.2	1.2	1.0	1.3	1.8	1.7	1.6	1.9	2.3	2.1
	Pex5	Zfand6	Idh1	1.1	1.0	1.0	1.0	1.1	1.1	1.1	1.0	1.0	1.6	1.8	1.7	1.6	2.0	1.6
	Tysnd1	Tmem135	Lonp2	1.1	1.1	1.0	1.1	1.1	1.3	1.2	1.1	1.2	1.6	1.9	1.9	1.8	2.5	1.7
Mitochondrial β-oxidation	Hmgcs2	Cpt2	Acsl1	2.8	1.8	1.8	3.6	1.9	2.4	1.9	1.9	1.7	1.6	2.1	2.5	1.6	3.3	2.2
	Etfdh	Decr1	Acot2	1.5	1.6	4.5	1.7	2.4	5.8	1.7	1.7	7.7	1.8	2.1	9.2	2.1	4.4	16.3
	Pdk4	Acadl	Hadha	11.4	1.3	1.4	12.2	1.8	2.3	2.5	1.5	1.8	168.1	1.6	1.9	109.2	4.1	3.4
	Acad11	Crat	Acacb	1.3	1.3	1.4	1.7	1.4	1.6	1.7	1.6	1.7	1.8	5.4	3.1	2.3	8.5	6.0
	Cpt1a	Eci2	Acsl5	1.8	1.9	1.0	1.8	2.4	1.1	2.0	1.8	1.2	1.1	1.4	1.7	2.2	4.2	2.6
	Cox6b2	Eci1	Acadm	1.0	1.3	1.0	1.6	2.1	1.2	1.0	1.1	1.1	1.7	2.0	1.9	6.2	4.4	3.4
Mitochondrial carriers	Slc25a34	Slc25a20	Slc25a42	1.9	1.6	1.7	1.8	2.0	1.6	1.7	1.6	2.2	1.9	2.4	2.8	1.8	4.1	2.8
	Ucp2	Slc25a32	Slc25a46	1.3	1.1	1.1	2.6	0.0	1.0	2.1	1.0	1.1	0.0	1.9	1.6	6.3	2.5	1.7
	Slc25a30			1.3			1.2			1.6			3.6			3.6		
Other mitochondrial functions	Gpam	Gpcpd1	Timm9	1.3	1.0	1.2	1.0	1.0	1.0	2.0	1.7	1.0	2.2	1.6	2.1	1.6	4.9	1.7
	Chchd4	Grpel2	Chdh	1.1	1.4	1.0	1.0	1.2	1.3	1.0	1.1	1.0	1.7	3.0	1.6	1.8	3.1	1.8
	Gpd1	Mfn2	Gpd2	1.0	1.2	1.0	1.2	1.1	1.0	1.3	1.2	1.2	1.9	1.6	3.3	1.6	2.1	2.2
	Aco2			1.0			1.0			1.0			2.4			3.3		
Microsomal FA oxidation	Cyp4a14	Cyp4a31	Aldh3a2	2.0	2.0	1.3	23.5	7.0	1.8	2.0	12.5	2.4	9.5	8.9	4.8	37.1	86.4	13.6
	Cyp4a10			1.2			13.4			1.5			3.7			8.1		
Glycogenolysis & gluconeogenesis	Fbp2	Ppp1r3g	G6pc	2.5	1.2	1.7	7.5	1.2	2.5	3.1	2.3	1.6	2.3	8.1	1.2	9.0	13.9	-1.2
	Gyk	Crls1	Slc16a1	0.0	1.0	1.1	1.3	1.0	1.2	1.3	1.1	1.1	2.0	1.9	2.7	1.7	1.8	2.6
Carnitine transporter	Slc22a5			2.5			2.8			3.3			6.1			22.2		
Retinoid metabolism	Retsat	Adh7		1.3	1.0		3.9	1.2		2.1	1.5		2.2	1.8		3.7	1.2	
Regulation of metabolism	Lepr	Adipor2	Fgf21	1.1	1.1	1.2	1.4	1.2	1.5	2.8	1.1	1.0	1.6	2.1	3.7	3.2	1.8	3.6
	Me1			1.1			1.1			2.1			6.8			4.6		
Inhibition of TG lipases activity	G0s2	Angptl4		5.2	2.2		5.1	2.2		2.1	1.8		6.8	1.8		4.6	3.5	
Lipid mobilisation	Acot1	Mgll	Pnpla2	32.8	1.7	1.2	44.1	3.1	1.3	10.6	2.8	1.4	15.9	2.3	1.7	55.5	7.1	2.2
	Lipg	Lipe	Lipa	1.0	1.6	1.0	1.0	2.3	1.3	1.0	1.5	1.3	3.4	1.5	1.6	2.2	2.8	1.7
	Pla2g6	Lpin2		1.1	1.2		1.0	1.2		1.4	1.4		3.1	2.4		3.2	3.7	
Lipid storage	Cidec	Plin5	Hilpda	1.5	1.9	2.3	2.6	2.4	1.9	2.4	2.3	1.9	12.2	3.1	3.6	27.0	5.3	1.5
	Fitm1	Plin4	Agpat9			1.5	1.2		1.4	1.1	1.2	1.4	2.1	2.3	2.5	5.5	2.4	3.1
	Agpat6	Ptpla	Plin2		1.6		[illegible]	1.4	1.0	[illegible]	1.4	1.1	2.3	2.0	2.5	2.1	3.5	4.4
Intracellular oxysterols receptors	Osbpl11	Osbpl3		1.1	1.0		1.0	1.0		1.0	1.0		1.7	3.3		1.7	2.7	
Cholesterol & sex hormons biosynthesis	Hsd17b7	Hsd17b12	Hsd17b11	1.3	1.0	1.0	1.3	1.0	1.7	*1.6*	1.0	1.5	2.3	1.7	3.8	2.4	3.4	8.2
Synthesis of lipid precursors	Mogat1			1.4			1.9			1.1			5.7			32.1		
Lipids transporters and carriers	Fabp4	Fabp1	CD36	1.0	1.4	1.2	1.3	8.0	2.8	2.6	1.5	1.7	2.8	1.3	3.6	76.8	1.6	36.8
	Mttp			1.1			1.3			1.5			1.9			2.1		
Lipoproteins	A1cf	Vldlr		1.0	1.1		1.0	1.1		1.3	-1.2		4.2	1.6		2.3	5.7	
Transcription factors	Klf10	Txnip	Creb3l3	1.9	3.8	2.1	1.7	3.1	2.9	1.3	1.5	3.0	3.0	1.5	1.3	2.0	-1.2	1.6
	Sp3	Jun	Rbpms	1.1	1.1	1.1	1.2	1.0	1.1	1.0	1.0	1.1	2.1	2.2	1.8	1.7	1.9	1.6
	Nr1i2	Ubp1	E2f8	1.1	1.1	1.0	1.1	1.1	1.0	1.2	1.4	1.0	1.8	1.7	3.2	2.0	1.8	4.8
