# Supplementary material for: Transcriptomic signatures of peroxisome proliferator-activated receptor α (PPARα) in different mouse liver models identify novel aspects of its biology
Source: BMC Genomics. 2014 Dec 15;15(1):1106. doi: 10.1186/1471-2164-15-1106 (PMC4378209; doi:10.1186/1471-2164-15-1106)
Supplement: Supplementary file 3 — Additional file 3: PPARα tailored gene functional analysis (upregulation). Significantly upregulated genes that were overlapping between at least two hepatic models were selected for a functional analysis using an open access database GeneCards. Based on the provided information, the selected genes were grouped into functional categories and analyzed in each hepatic model i.e. primary hepatocytes treated with Wy14643 for 6h or 24 h (PH 6H or PH 24 h), precision cut liver slices treated with Wy14643 for 24 h (PCLS 24 h), and livers of mice treated with Wy14643 for 6 h or 5 days (L6h or L5d). Genes that were significantly altered are depicted in bold and underlined, red color indicates upregulation, black no change, and green downregulation of gene expression. Fold change was calculated as the average gene expression value in treatment group vs. control. (PDF 391 KB) [file 12864_2014_6870_MOESM3_ESM.pdf]

Additional file 3: PPARα tailored gene functional analysis (upregulation).

Biological function	Genes			PH6h			PH 24h			PCLS 24h			L 6h			L 5d		
ECM	Angptl4	Lgals4	St3gal5	2.2	2.9	1.8	2.2	2.7	2.4	1.8	2.7	2.1	1.8	3.6	3.1	3.5	7.2	6.3
	Vnn1	Bace1	Inhbe	1.8	1.3	1.3	3.4	1.2	1.4	2.0	1.7	2.6	9.6	2.6	1.9	61.2	2.0	1.2
	Lamb3	Sorbs1	Adamts15	1.4	1.0	[illegible]	3.6	1.1	1.1	1.1	1.0	[illegible]	13.0	2.1	1.8	30.5	1.9	2.3
	Alpl	Hyal2	Optn	1.6	1.2	1.1	1.9	1.1	1.2	1.6	1.1	1.1	3.7	1.6	1.6	4.1	1.7	2.2
	Galnt2	Hyal1	Pvrl2	1.1	1.3	1.1	1.2	1.3	1.1	1.1	1.3	1.0	1.6	1.6	1.8	2.5	1.7	2.3
	Igsf5	Tfpi2	Igsf11	1.2	[illegible]	1.1	1.1	1.4	1.2	1.1	-1.5	1.3	1.7	1.7	1.9	1.7	1.9	2.9
	L3hypdh	Spon2	Arl4a	[illegible]	[illegible]	1.1	1.1	1.1	1.0	1.2	1.5	1.2	1.9	1.9	3.0	1.7	1.9	2.9
	Fbf1	Plat	Spp1	1.1	2.7	[illegible]	1.0	2.5	[illegible]	[illegible]	1.4	[illegible]	1.8	[illegible]	[illegible]	2.0	[illegible]	-1.2
Cytoskeleton	Krt23	Myom1	Baiap2l1	1.1	1.5	1.3	1.1	1.2	1.2	1.6	1.3	1.3	5.0	2.6	2.0	18.0	1.6	3.8
	Slk	St5	Capn2	1.1	1.3	1.0	[illegible]	1.1	1.0	[illegible]	-1.2	1.1	1.9	1.8	1.6	2.0	1.8	1.7
	Csrp3	Krt79	Larp4	[illegible]	[illegible]	1.1	1.2	1.2	1.0	[illegible]	1.0	1.1	1.7	1.7	1.6	2.4	3.6	1.7
	Ssh2	Eps8	Pkp2	1.1	[illegible]	1.1	[illegible]	1.1	1.1	1.1	[illegible]	[illegible]	1.6	3.1	2.1	2.1	3.8	2.1
	Rhobtb1			[illegible]			[illegible]			1.1			1.8			1.7		
Endothelial functions	Adtrp	Lipg	Angptl4	1.5	[illegible]	2.2	2.0	1.0	2.2	1.6	[illegible]	1.8	1.8	3.4	1.8	2.8	2.2	3.5
	Nrp2	E2f8		1.1	[illegible]		[illegible]	1.0		[illegible]	1.5		3.0	3.2		3.9	4.8	
Immunity	Mmd	Ly6d	Ctse	1.7	1.1	1.1	2.1	2.0	1.8	1.5	2.1	1.6	2.2	1.8	3.2	2.6	2.5	9.0
	Ifi202b	Hilpda	Abcb4	1.1	2.3	1.4	1.2	1.9	1.8	[illegible]	1.9	1.6	1.2	3.6	1.4	-1.3	1.5	1.9
	Cblb	Il1rn	Csf2rb	1.1	[illegible]	1.1	1.0	[illegible]	1.1	1.1	-1.4	[illegible]	1.8	4.4	2.3	1.9	4.1	10.3
Integrity of Golgi	Rab30	Caln1	Dnmbp	2.0	1.1	1.1	2.3	1.0	[illegible]	2.8	1.5	1.3	7.0	1.6	2.5	18.2	1.5	2.5
	Tgoln1	Sar1b	Ddhd2	1.1	1.1	1.3	1.1	1.3	1.4	1.1	1.3	1.5	3.0	1.7	3.2	2.6	1.6	3.2
	Rab9	Atp6v0a2	Tmed5	1.8	[illegible]	1.1	1.5	[illegible]	1.3	1.4	[illegible]	1.2	2.3	1.6	2.6	2.6	2.5	4.7
Vesicular trafficking	Pctp	Sgk2	Pllp	1.3	2.0	1.2	1.8	1.7	1.3	2.1	1.7	1.2	7.3	2.1	1.5	8.3	1.5	2.0
	Stk16	Mrap	Rab43	1.2	1.1	[illegible]	1.1	1.1	1.1	1.1	1.3	1.1	1.7	1.8	1.5	1.7	2.6	1.9
	Myo5b	Chpt1		1.2	[illegible]		1.2	1.4		1.1	[illegible]		1.6	1.8		1.6	3.5	
Endocytosis	Rin2	Ehd4	Arl8b	1.3	[illegible]	1.1	1.3	1.0	1.0	1.8	1.1	[illegible]	2.5	2.3	1.7	2.3	3.4	2.7
	Pdzrn3	Trak1	Picalm	1.2	1.1	1.1	1.0	1.1	1.0	-2.4	1.0	1.2	1.5	1.6	2.4	5.3	1.7	2.2
	Mtm1	Plekha8	Lrp4	1.0	1.1	1.1	[illegible]	1.1	1.2	1.1	1.1	1.4	2.0	2.0	1.9	2.6	1.7	1.9
Neurotransmission	Qpct	Tenm3	Trpv2	1.3	1.4	1.0	1.6	1.7	1.2	1.6	1.7	[illegible]	2.1	2.2	6.3	6.4	3.4	11.5
	Enc1	Chrna2	Arsa	1.2	1.2	1.0	1.2	2.3	1.1	1.1	1.6	1.1	4.5	3.4	1.9	3.6	8.2	2.3
	Clstn3	Nln	Synpo	[illegible]	1.1	1.1	[illegible]	1.1	1.1	1.2	1.2	1.2	3.5	3.1	2.4	5.6	4.9	3.1
	Snrk	Slc6a9	Gmfb	1.3	1.1	1.1	1.2	1.0	1.0	1.2	1.2	1.0	1.9	1.9	1.7	1.7	1.8	1.6
	Rtn4	Slc22a3	Sema5b	1.1	1.0	[illegible]	1.1	1.1	[illegible]	[illegible]	1.1	1.0	1.7	1.6	1.6	3.4	2.8	2.4
	Adcy6	Mtnr1a	Unc119	1.1	-1.2	1.1	1.1	[illegible]	[illegible]	[illegible]	1.0	1.2	1.8	1.8	1.9	2.3	4.4	3.4
Apoptosis	Peg3	Tgm2	Bcl2l13	[illegible]	1.1	1.1	1.1	1.1	1.0	2.1	[illegible]	1.0	2.3	1.9	1.9	6.8	1.8	1.9
	Stk17b	Fas	Dram2	[illegible]	[illegible]	[illegible]	[illegible]	1.2	1.1	[illegible]	-1.3	1.1	1.5	2.4	1.9	2.6	2.1	2.0
	Aifm2	Casp8	Bfar	1.1	1.3	[illegible]	1.1	1.3	1.0	1.2	1.2	1.0	2.6	2.2	1.9	3.7	2.6	2.4
	Bag4	Arel1		[illegible]	1.0		[illegible]	[illegible]		[illegible]	1.1		1.6	2.4		1.8	2.4	
Cell cycle	Ccnd1	Wee1	S100a10	-1.2	[illegible]	[illegible]	[illegible]	1.0	[illegible]	1.1	1.3	1.1	1.8	1.8	1.5	2.2	3.4	1.7
	Usp10	Gadd45b	Cdkn1a	1.1	1.1	1.1	1.1	1.3	[illegible]	1.1	-1.4	1.1	1.7	9.8	5.1	1.7	6.5	2.3
	Ccng2	Aptx	Prkdc	1.1	1.0	[illegible]	1.1	1.0	[illegible]	[illegible]	[illegible]	1.2	3.8	2.0	2.4	4.2	4.3	6.1
	Sycp3	Spc25	Nek2	1.2	1.1	1.1	1.1	1.2	1.0	[illegible]	1.5	[illegible]	1.9	1.9	1.6	2.1	7.0	5.5
	Tacc2	Xrcc3	Celf2	1.7	[illegible]	1.1	1.6	1.0	1.1	1.1	1.1	[illegible]	1.5	1.6	2.2	2.8	2.0	2.2
	Ctif	Gemin2	Pus10	1.3	1.1	1.1	1.3	1.2	[illegible]	1.6	1.1	1.0	1.7	2.2	2.0	2.3	3.7	1.6
	Thumpd3	Khdrbs3		1.0	1.1		[illegible]	1.0		1.1	1.1		1.9	1.9		2.4	2.3	
