# Supplementary material for: Transcriptomic signatures of peroxisome proliferator-activated receptor α (PPARα) in different mouse liver models identify novel aspects of its biology
Source: BMC Genomics. 2014 Dec 15;15(1):1106. doi: 10.1186/1471-2164-15-1106 (PMC4378209; doi:10.1186/1471-2164-15-1106)
Supplement: Supplementary file 4 — Additional file 4: PPARα tailored gene functional analysis (downregulation). Significantly downregulated genes that were overlapping between at least two hepatic models were selected for a functional analysis using an open access database GeneCards. Based on the provided information, the selected genes were grouped into functional categories and analyzed in each hepatic model i.e. primary hepatocytes treated with Wy14643 for 6 h or 24 h (PH 6H or PH 24 h), precision cut liver slices treated with Wy14643 for 24h (PCLS 24 h), livers of mice treated with Wy14643 for 6 h or 5 days (L6h or L5d). Genes that were significantly altered are in bold and underlined, red color indicates upregulation, black no change, and green downregulation. Fold change was calculated as the average gene expression value in treatment group vs. control. (PDF 430 KB) [file 12864_2014_6870_MOESM4_ESM.pdf]

Additional file 4: PPARα tailored gene functional analysis (downregulation).

Biological function	Genes			PH6h			PH 24h			PCLS 24h			L 6h			L 5d		
Transcriptional co-repression	Klf12	Snai2	Hhex	-1.2			-1.1			-1.6			-1.7	-2.7	-2.9	-2.0	-1.6	-2.0
	Nab2	Irf2	Tsc22d1	-1.1		1.2	-1.2		1.1	-1.2	-1.1	1.3	-2.3	-2.3	-2.1	-2.7	-2.5	-2.7
	Sirt7	Nmi	Id2			-1.1						1.1	-1.9	-1.7	-2.0	-2.0	-2.0	-3.3
Transcriptional activation	Foxa3	Parp14	Atf6b				-1.2	1.1	-1.1	-1.4			-1.8	-2.0	-1.9	-1.5	-2.2	-1.7
	Camk1d	Myrf	Xbp1				-1.1						-1.7	-1.6	-1.9	-3.5	-1.6	-2.0
	Irf6	Foxa2	Mlx		-1.1					1.0	1.0		-1.6	-1.8	-1.7	-2.0	-1.8	-1.6
NF-κB (downstream activation)	Tifa	Egfr	Tnfsf12				-1.1	-1.1		-1.8	-1.2	-1.3	-3.0	-2.6	-2.3	-1.5	-3.9	-1.7
	Ufl1	Cxxc5	Ikbke			-1.1			-1.2			-1.4	-1.8	-1.6	-1.6	-1.8	-2.3	-2.9
Coagulation	F11	Avpr1a	Pde4b	-1.1			-1.2		-1.1	-1.6		-1.4	-1.7	-3.7	-2.9	-6.6	-4.1	-4.7
	Serpina10	Serpinf2	F2r	-1.2	-1.1	-1.2	-1.3	-1.2	-1.1	-1.2		1.1	-2.3	-1.8	-1.9	-1.8	-2.8	-2.2
	Klkb1	Serpina9	F7				-1.4		-1.1				-2.0	-1.8	-1.6	-2.3	-3.6	-5.2
Acute phase	Orm2	Orm3	Crp		-1.1				-1.3	-1.7	-1.7	-1.6	-5.2	-2.4	-2.2	-2.8	-4.1	-2.3
	Saa1	Saa2	Saa4		-1.1	-1.1		1.0	-1.2	-1.5	-1.2	-1.3	-15.1	-68.7	-3.7	-8.3	-1.9	-3.1
	Ier2			1.0									-2.4			-3.4		
Immunity	Il1r1	Nostrin	Il13ra1	-1.1			-1.2		1.0	-1.9	-1.2		-2.7	-2.3	-2.2	1.3	-2.2	-2.2
	Irgm2	Cish	Grem2	1.0	-1.1	-1.2	1.2	-1.1	1.3		-1.2		-1.9	-2.4	-1.7	-1.6	-2.8	-1.8
	Il18	Ly96	Sema4a		1.1	-1.1	-1.1	-1.1					-1.6	-1.6	-1.6	-3.0	-1.7	-1.6
	Ddx58	Rnf144b	Nfil3				1.1	-1.1	1.0	1.2			-1.6	-1.5	-4.3	-1.8	-3.1	-3.1
	Ifih1	Cxcl1					1.1	-1.2			-1.3		-1.6	-9.2		-2.2	-1.7	
T cells functions	Zap70	Alcam	Il18						-1.1		-1.2		-1.9	-1.9	-1.6	-3.8	-1.9	-3.0
	Sema4a	Nfil3	Ifih1	-1.1				1.0	1.1				-1.6	-4.3	-1.6	-1.6	-3.1	-2.2
	Xbp1												-1.9			-2.0		
B cells functions	Zap70	Alcam									-1.2		-1.9	-1.9		-3.8	-1.9	
Angiogenesis	Lect1	Nostrin	Tnfsf12								-1.2	-1.3	-1.8	-2.3	-2.3	-2.6	-2.2	-1.7
Renin-angiotensin system	Serpina3m	Serpina3n					-1.4	-1.2		-1.6	-1.3		-3.7	-2.8		-6.0	-2.0	
ECM	Prg4	Fgfr4	Cdh1		1.1	1.0		1.1		-1.5	-1.4	1.1	-3.1	-2.5	-2.3	-4.0	-2.6	-3.9
	Sorl1	Myh10	Slc9a3r1									-1.2	-2.2	-2.2	-2.1	-2.2	-1.7	-2.2
	Fndc4	S1pr1	Lgals8									1.0	-1.8	-1.8	-1.7	-2.2	-2.2	-1.9
	Omd	Sgms2	Arhgap26	-1.2	1.1		-1.6		1.0	1.1	1.0		-1.7	-1.7	-1.6	-2.0	-2.0	-1.6
Cytoskeleton	Fmn2	Lrrc16a	Lasp1	-1.3		1.0				-1.2			-3.7	-3.5	-2.2	-4.2	-3.2	-1.9
	Neb	Myo1b	Shroom1	1.1		1.1		-1.2	1.0		-1.2		-2.3	-2.1	-2.1	-3.3	-3.7	-2.7
	Frmd4b	Inf2	Ehbp1	1.1	1.0					1.0			-2.1	-1.9	-1.7	-1.7	-1.7	-1.9
	Cth						-1.2			1.0			-1.6			-2.5		
Tight junctions	Cldn1	Marveld2					-1.2						-2.5	-2.1		-4.7	-1.6	
Transporters	Slc41a2	Atp8b1	Slc17a2	1.0				-1.2	-1.3			-1.7	-9.3	-3.2	-1.3	-4.3	-3.0	-1.8
	Slco1a4	Slc3a1	Slc29a1	-1.3	-1.3		-1.3	-1.8			1.1		-2.6	-2.4	-2.1	-8.6	-3.8	-2.2
	Abcc6	Slc36a1	Slc25a15		1.0								-2.1	-2.1	-2.0	-2.9	-2.8	-1.8
	Slc39a14	Slc17a3	Slc13a3		-1.5			-1.5	-1.2	-1.2	-1.4		-2.5	-1.6	-1.6	-1.9	-2.7	-2.4
Vesicular trafficking	Rab4b	Rab4a	Sec23b								1.1		-2.9	-1.9	-2.1	-1.5	-1.9	-1.8
	Myo1b	Sorl1					-1.2			-1.2			-2.1	-2.2		-3.7	-2.2	
Metabolism of aa	Sds	Prodh	Cdo1					-1.2	-1.3	-1.5	1.1	-1.5	-5.0	-2.0	-1.9	-1.7	-4.9	-2.5
	Gldc	Gls2	Bcat2		1.1		-1.2	1.1					-1.9	-1.8	-2.3	-2.4	-9.2	-2.3
	Cbs						-1.4			-1.2			-1.7			-3.5		
Glycogen metabolism	Ppp1r14a	Slc37a4		1.1	-1.2		-1.2	-1.3					-7.7	-2.2		-8.0	-2.2	
Steroid metabolism	Hsd3b2	Hsd3b5						1.1		1.1	1.2		-3.9	-4.7		-14.7	-72.5	
Lipogenesis	Thrsp	Mid1ip1	Acly		1.1	1.1				1.1	1.0		-3.4	-2.4	-1.6	-5.3	-1.9	-2.4
	Nudt7			1.2			1.3			1.4			-2.1			-1.8		
Phase I &II of drug metabolism	Cyp2b10	Cyp2c70	Cyp2u1	-1.2		1.0	-1.6	-1.2		-1.9	-1.4	1.1	-2.4	-1.7	-1.6	-4.4	-11.4	-2.8
Neurotransmission	Fam134b	Hs6st1	Kcnn2		1.0	-1.4			-1.2				-2.1	-1.8	-1.8	-1.7	-1.7	-2.1
	Pnpla6	Manf	Aldh1b1		-1.2	1.0		-1.2	1.0		-1.2	1.4	-1.9	-2.5	-1.7	-1.7	-2.3	-3.8
	Gfra1	Ttll1	Myrf				-1.6	1.1					-1.8	-1.7	-1.6	-1.8	-1.6	-1.6
	Shroom			1.1			1.0						-2.1			-2.7		
